# Atypical retrobulbar optic neuropathy after semaglutide escalation

**DOI:** 10.1210/jcemcr/luag108

**Published:** 2026-05-29

**Authors:** Shivaprasad Channabasappa, Riddhi Das Gupta, Vidhya Chandran, Sai Prasad

**Affiliations:** Department of Endocrinology, Sapthagiri Institute of Medical Sciences and Research Centre, Bengaluru 560090, India; Department of Endocrinology, Sapthagiri Institute of Medical Sciences and Research Centre, Bengaluru 560090, India; Department of Ophthalmology, Sankara Eye Hospital, Bengaluru 560037, India; Department of Internal Medicine, S. Nijalingappa Medical College and HSK Hospital and Research Centre, Bagalkot 587102, India

**Keywords:** semaglutide, optic neuropathy, NAION, retrobulbar optic neuropathy, GLP-1 receptor agonist, obesity

## Abstract

Semaglutide, a glucagon-like peptide-1 receptor agonist (GLP-1RA), is widely used to manage type 2 diabetes and obesity. Recent pharmacovigilance signals have reported an increased incidence of nonarteritic anterior ischemic optic neuropathy (NAION) among semaglutide users, although the absolute risk remains low. We report a man in his early 30s with class III obesity, obstructive sleep apnea, and prediabetes who developed acute, painless, asymmetric bilateral visual dysfunction four weeks after semaglutide escalation to 1 mg per week. Evaluation revealed retrobulbar optic neuropathy with asymmetric visual field defects, a central scotoma in the left eye, an altitudinal defect in the right eye, preserved optic disc appearance, and markedly delayed visual evoked potentials. Optical coherence tomography and neuroimaging were unremarkable. The presentation was atypical for NAION but did not fully align with classical demyelinating optic neuritis, yielding a mixed clinical picture. Semaglutide was discontinued immediately, and visual function remained stable over serial follow-up with adaptation to a persistent left central scotoma but no further deterioration. This case illustrates an atypical retrobulbar optic neuropathy in close temporal proximity to semaglutide dose escalation and underscores the need for clinical vigilance and strengthened pharmacovigilance as GLP-1RA use expands globally.

## Introduction

Glucagon-like peptide-1 receptor agonists (GLP-1RA) have become central to the management of type 2 diabetes and obesity, driven by their potent effects on weight reduction, glycemic control, and cardiometabolic risk reduction. Among them, semaglutide has seen rapid global uptake, including for obesity treatment, where its efficacy has led to widespread clinical and public interest.

Although GLP-1 receptor agonists (GLP-1RA) are widely used for type 2 diabetes and obesity, emerging evidence has raised concerns about their ocular safety [1]. Over the past two years, case reports have described optic neuropathies temporally associated with semaglutide use, including presentations with visual field defects, disc edema, and acute visual loss [2, 3]. Proposed mechanisms include altered optic nerve head perfusion and vascular autoregulation changes [4, 5]. Interactions with pre-existing anatomical susceptibility factors, including a crowded optic disc configuration, may further amplify this risk [1, 4]. Observational cohort and registry studies have subsequently reported an increased incidence of NAION among semaglutide users compared with other antidiabetic therapies [6, 7]. Additional large population-based analyses have corroborated these signals, although the absolute incidence remains low, and most treated individuals do not develop optic neuropathy [8-10]. A medical product alert issued by the World Health Organization highlighted this potential risk [11], and the European Medicines Agency has classified NAION as a rare adverse effect requiring inclusion in product information [12].

We describe a young adult who developed acute retrobulbar optic neuropathy shortly after semaglutide dose escalation. The clinical picture demonstrated overlapping features of ischemic and demyelinating optic neuropathies without the classical findings of either condition, highlighting the diagnostic challenges posed by atypical optic neuropathies and the need for strengthened pharmacovigilance as GLP-1RA use continues to grow.

## Case presentation

A man in his early 30s with class III obesity (body mass index 47 kg/m^2^), severe obstructive sleep apnea managed with continuous positive airway pressure therapy, prediabetes, and dyslipidemia presented for medical management of obesity after unsuccessful lifestyle interventions. Semaglutide was initiated at 0.25 mg once weekly and titrated in standard 4-week increments to 0.5 mg and then 1 mg weekly. Over three months, weight reduction was steady (approximately 8% of baseline) without rapid glycemic change (HbA1c 6.4% [46 mmol/mol] to 6.3% [45 mmol/mol]). He received structured counselling on diet (target caloric deficit of 500-750 kcal/day), physical activity (aiming for at least 150 minutes/week of moderate-intensity exercise), and sleep hygiene while continuing continuous positive airway pressure therapy, with moderate adherence and a tendency for weight loss to plateau after 12 weeks.

Four weeks after escalation to 1 mg, he awoke with painless, predominantly left-sided visual blurring and described brownish halos around digital screens. There was no ocular pain on movement, photophobia, diplopia, flashes, floaters, or symptoms of retinal detachment. He denied preceding viral illness or systemic symptoms. There was no exposure to medications associated with optic neuropathy, including amiodarone, phosphodiesterase-5 inhibitors, ethambutol, isoniazid, linezolid, or chemotherapeutic agents. He presented promptly for evaluation due to concern regarding acute, unexplained visual changes.

## Diagnostic assessment

Best-corrected visual acuity was 6/6 in the right eye and N6 for near vision. The left eye demonstrated reduced near vision (N8) and distance visual acuity in the left eye could not be reliably recorded due to central blurring. Pupillary reactions were normal bilaterally, although a relative afferent pupillary defect was present in the left eye. Intraocular pressures were within normal limits. Fundus examination revealed healthy-appearing optic discs in both eyes, with a cup-to-disc ratio of 0.3 and no disc edema. (Table 1)

**Table 1:** Ophthalmic findings of our patient

Parameter	Right Eye (OD)	Left Eye (OS)
Best-corrected visual acuity	6/6 (distance), N6 (near)	**Distant Vision Not Recorded**, N8 (near)
Pupillary reactions	Normal direct & consensual reflexes	Normal direct & consensual reflexes
Relative afferent pupillary defect (RAPD)	Absent	**Present**
Intraocular pressure (IOP)	Within normal limits	Within normal limits
Fundus examination	Healthy optic disc, C/D ratio 0.3, no edema	Healthy optic disc, C/D ratio 0.3, no edema
Visual fields (HFA 30-2)	**Superior defect**	**Central scotoma**
Color vision (Ishihara)	21/21 plates correct	**3/21 plates correct**
Contrast sensitivity (digital CS)	**2.10 (reduced)**	**1.65 (reduced)**
Optical coherence tomography (OCT)	Normal ONH and RNFL	Normal ONH and RNFL
Visual evoked potentials (VEP)	**Reduced amplitude, increased latency**	**Reduced amplitude, increased latency**
MRI brain/orbits	No abnormalities	No abnormalities

Abnormal parameters are highlighted in bold.

Abbreviations: C/D, cup-to-disc ratio; CS, contrast sensitivity; HFA, Humphrey Field Analyzer; MRI, magnetic resonance imaging; OD, right eye (oculus dexter); ONH, optic nerve head; OS, left eye (oculus sinister); RNFL, retinal nerve fiber layer.

**Figure 1:**
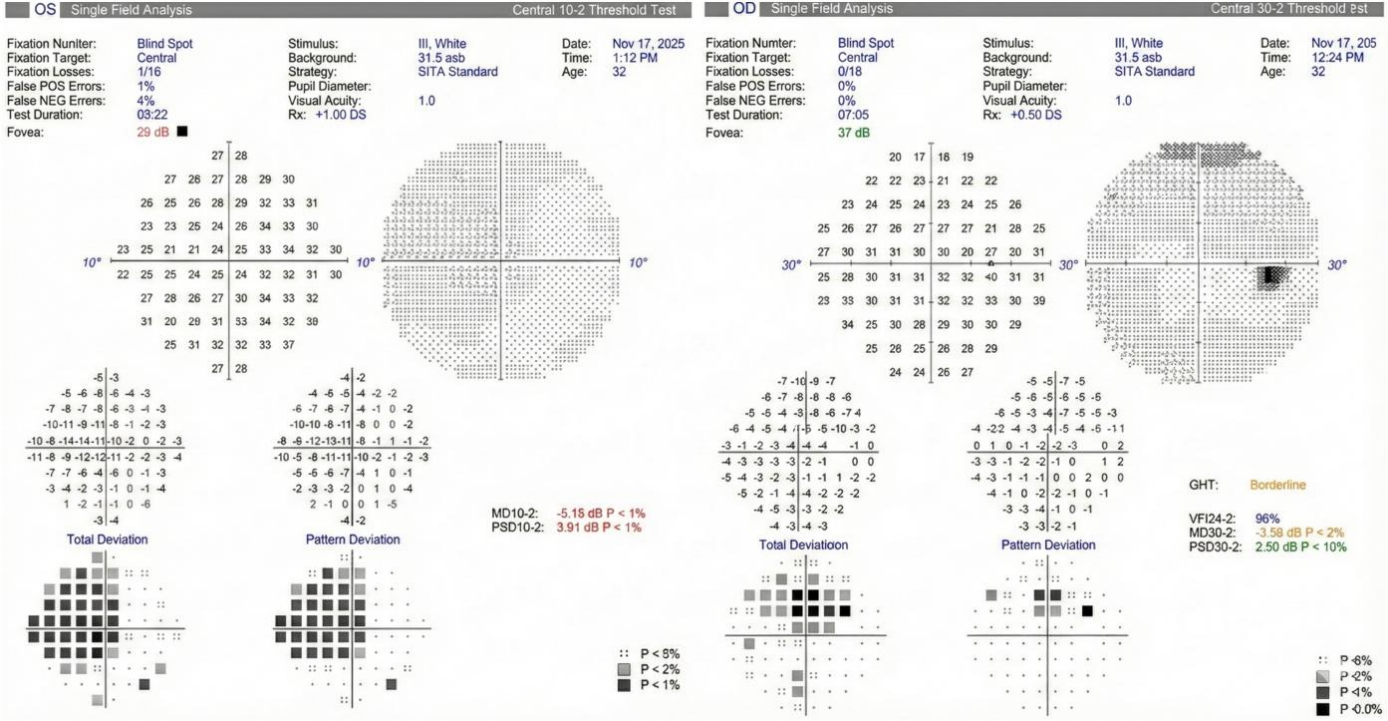
Humphrey field analyzer (HFA 30-2) visual field plots demonstrating asymmetric bilateral visual field defects. The right eye shows a superior defect, while the left eye demonstrates a central scotoma. These findings are consistent with functional impairment of the optic nerve, with left-predominant involvement.

**Figure 2:**
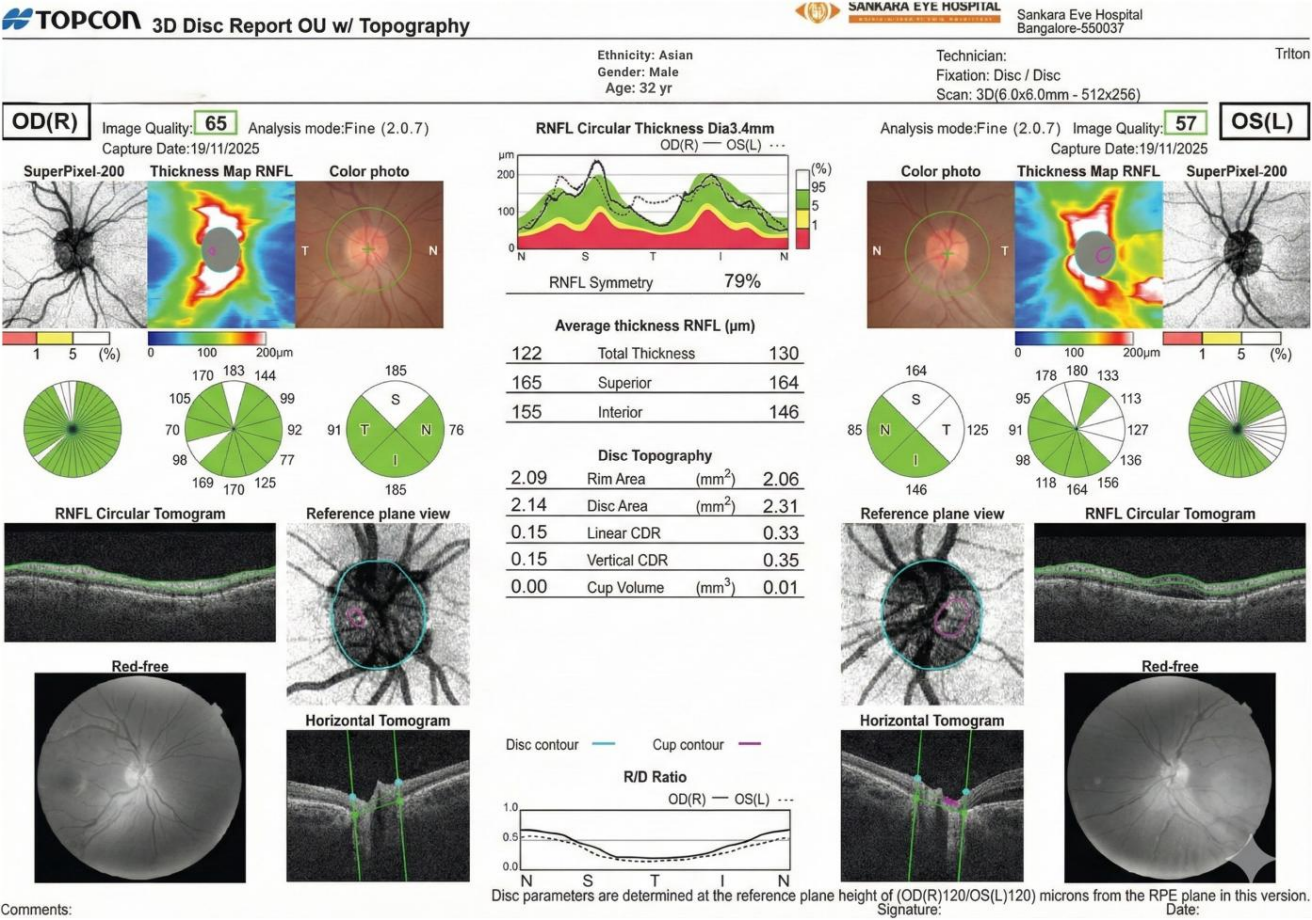
Optical coherence tomography (OCT) images of the optic nerve head (ONH) and retinal nerve fiber layer (RNFL) demonstrating normal structural appearance in both eyes. The absence of optic disc edema or RNFL thinning highlights the retrobulbar nature of the optic neuropathy and the discordance between structural and functional findings.

**Figure 3:**
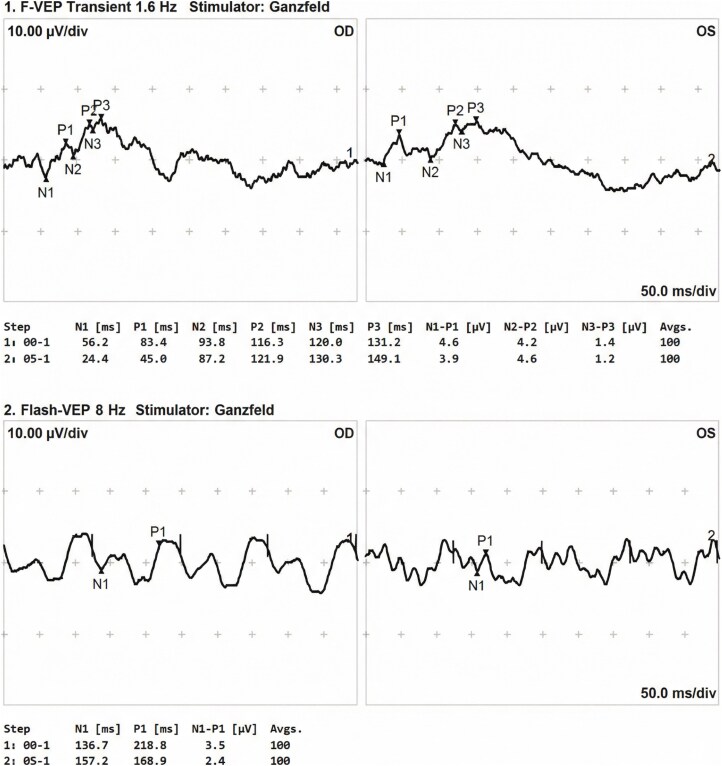
Visual evoked potentials (VEP) showing reduced amplitude and markedly increased P100 latency in both eyes. VEP abnormalities indicate delayed conduction along the visual pathways, supporting the diagnosis of retrobulbar optic neuropathy despite normal optic disc appearance on examination. A Ganzfeld stimulator provides uniform, full-field illumination to elicit global retinal responses during electrophysiological testing.

Automated perimetry (Humphrey Field Analyzer) showed a superior defect in the right eye and a central scotoma in the left eye. (Figure 1) Color vision was intact in the right eye (21/21 Ishihara plates) but markedly reduced in the left eye (3/21 plates), mirroring the asymmetry in visual acuity and field loss. Contrast sensitivity was decreased bilaterally (2.10 OD, 1.65 OS). Optical coherence tomography (OCT) of the optic nerve head demonstrated normal retinal nerve fiber layer thickness without evidence of swelling or thinning. (Figure 2) Visual evoked potentials revealed reduced amplitudes and significantly delayed P100 latencies in both eyes, consistent with retrobulbar optic nerve dysfunction. (Figure 3) A Ganzfeld stimulator, which provides uniform full-field illumination to elicit global retinal responses, was used during electrophysiological testing. Magnetic resonance imaging of the brain and orbits, including dedicated orbital sequences, showed no abnormalities and no optic nerve enhancement. Laboratory evaluation, including inflammatory markers, vitamin B12, and thyroid function tests, was within normal limits.

## Treatment

Semaglutide was discontinued because of the close temporal association between dose escalation and symptom onset, and in view of emerging reports linking GLP-1 receptor agonists with optic neuropathies. The patient was counselled regarding the suspected drug-related adverse event, the uncertainty surrounding its underlying mechanism, and the potential but unproven contribution of semaglutide to his optic neuropathy.

For optic neuropathies of uncertain etiology and in the absence of inflammatory or compressive features on imaging, there is no evidence-based pharmacological treatment that reliably reverses vision loss. Corticosteroids were avoided as they have no proven benefit in NAION, and imaging showed no inflammatory features. Accordingly, management focused on mitigating potentially modifiable contributors and preventing further optic nerve injury. His severe obstructive sleep apnea was reassessed, and adherence to continuous positive airway pressure therapy was reinforced, given its established association with optic nerve vulnerability and NAION risk. Cardiometabolic risk factors—including dyslipidemia, prediabetes, and class III obesity—were addressed through a non-GLP-1-based weight-management plan incorporating structured dietary guidance, physical-activity targets, and sleep optimization.

The ophthalmology team advised visual safety precautions, avoidance of nocturnal hypotension, and prompt reporting of any new visual symptoms. Corticosteroids or neuroprotective agents were not initiated, as imaging showed no inflammatory or compressive pathology, and there was no evidence to support their use in this context. A schedule for repeat visual field testing, contrast sensitivity assessment, and optic nerve evaluation was arranged.

## Outcome and follow-up

At short-term follow-up, the patient's visual function remained stable without further deterioration. He adapted to the persistent central scotoma in the left eye and reported no new visual symptoms. Repeat ophthalmic evaluation demonstrated unchanged visual fields, stable contrast sensitivity, and preserved visual acuity in the right eye, with no delayed optic disc changes or retinal nerve fiber layer thinning on serial optical coherence tomography.

Following discontinuation of semaglutide, the patient experienced modest weight regain of 3.2 kg over eight weeks despite continued adherence to lifestyle measures. Non-GLP-1 pharmacological options for weight management (including orlistat and metformin) were discussed in detail, but the patient preferred to continue with structured behavioral strategies alone. His obstructive sleep apnea management remained stable with good adherence to continuous positive airway pressure therapy. Given the uncertain etiology of the optic neuropathy and the potential risk of further visual compromise, he was scheduled for three-monthly ophthalmology follow-up with formal perimetry, contrast sensitivity testing, and optic nerve assessment, and longer-term outcomes are being monitored.

## Discussion

Optic neuropathies in young adults present a diagnostic challenge, particularly when clinical features do not conform to classical patterns. In this case, the combination of acute painless visual loss, normal optic disc appearance, asymmetric visual field defects, and markedly delayed visual evoked potentials (VEP) indicated a retrobulbar optic neuropathy, but the underlying etiology remained uncertain. The differential diagnosis included demyelinating, ischemic, and drug-related mechanisms, each supported by certain features yet contradicted by others.

A demyelinating optic neuropathy was an important consideration. Delayed P100 latency on VEP is a characteristic electrophysiological marker of demyelination, and although pain with eye movements is common, painless presentations are well recognized. The asymmetric involvement also aligns with typical demyelinating patterns and with disorders such as multiple sclerosis or myelin oligodendrocyte glycoprotein (MOG)–associated optic neuritis [13]. However, magnetic resonance imaging (MRI) of the brain and orbits showed no optic nerve enhancement or demyelinating lesions. More definitive investigations—including cerebrospinal fluid analysis and aquaporin-4 (AQP4) and MOG antibody testing—were not pursued because the patient's vision had stabilized and he declined invasive testing. These limitations constrain the ability to definitively exclude a demyelinating process, but the absence of pain, lack of MRI enhancement, and stable clinical course lowered the pretest probability for inflammatory optic neuritis.

An ischemic mechanism was also plausible given the patient's vascular risk factors, including class III obesity, obstructive sleep apnea, and dyslipidemia [4]. Obstructive sleep apnea, in particular, has been independently linked to NAION through mechanisms including nocturnal hypoxia and optic nerve head vascular dysregulation [14]. These comorbidities collectively elevated this patient's risk of NAION. Individual case reports have documented NAION in temporal association with semaglutide use [15-17]. Large observational and registry studies have since corroborated this signal, demonstrating a statistically significant elevation in NAION incidence among semaglutide-treated patients [6, 7]. Further multinational and database analyses have added to this evidence base [8, 9]. Regulatory bodies now formally acknowledge this potential risk [11, 12]. However, several features in this case were atypical for classical NAION: the absence of optic disc edema, the presence of a central rather than altitudinal defect (although central scotomas can occur, they are less common), and markedly delayed VEP latency [4]. These findings suggest that if ischemia contributed, it may have been part of a broader or overlapping process rather than a typical NAION phenotype. The lack of prior ophthalmologic records also prevented assessment of whether the patient had a “disc-at-risk” configuration, an important anatomical susceptibility factor [2].

The electrophysiological pattern in this patient provides additional nuance. Classical teaching suggests that NAION primarily causes a reduction in VEP amplitude with relatively modest latency change, whereas demyelinating optic neuritis often produces marked latency delay with variable amplitude reduction. Nonetheless, ischemic optic neuropathies can occasionally exhibit both reduced amplitude and delayed latency, particularly when retrobulbar segments are involved or in the context of severe vascular compromise [5]. The combined marked amplitude reduction and significant latency delay in this case therefore did not definitively distinguish between ischemic and demyelinating etiologies.

A drug-related optic neuropathy remained a key diagnostic consideration because of the close temporal association between semaglutide dose escalation and symptom onset, as well as emerging pharmacovigilance signals linking GLP-1RA with optic neuropathies [18]. Although a direct toxic mechanism has not been established, hypothesis-generating data suggest that semaglutide may alter optic nerve head perfusion or interact with vascular and anatomical susceptibility factors, thereby modifying optic nerve vulnerability in predisposed individuals [4, 5]. In this patient, semaglutide may have acted as an amplifier of underlying vascular risk rather than a sole causative agent.

Notably, most published reports of optic neuropathy have involved semaglutide rather than other GLP-1RA or GLP-1/glucose-dependent insulinotropic polypeptide (GIP) receptor agonists [1, 5]. Several factors may explain this pattern: semaglutide's higher potency, its greater weight-loss effect, its widespread global use, and the larger exposed population contributing to stronger pharmacovigilance signals [8, 19]. Under-recognition in association with other agents remains possible, and further research is needed to clarify whether this association is molecule-specific or reflects a broader class effect [9, 10].

Taken together, this case illustrates the diagnostic complexity of atypical optic neuropathies in the context of GLP-1RA therapy. The overlapping ischemic and demyelinating features, absence of classical NAION findings, and temporal relationship with semaglutide escalation underscore the importance of maintaining a broad differential diagnosis and ensuring prompt ophthalmologic evaluation in patients receiving GLP-1RA who report new visual symptoms. This atypical retrobulbar optic neuropathy occurred in close proximity to semaglutide dose escalation in a young adult with multiple vascular risk factors and was characterized by a central scotoma, markedly delayed visual evoked potentials, and normal optic disc appearance—features that do not conform neatly to either classical NAION or demyelinating optic neuritis. As the use of semaglutide continues to expand globally, clinicians should maintain vigilance for visual symptoms, facilitate early ophthalmologic assessment, and contribute to pharmacovigilance efforts to better define the spectrum, mechanisms, and risk modifiers of potential optic nerve involvement.

## Learning points

- Semaglutide use may be temporally associated with atypical optic neuropathies, including presentations that do not fulfil classical criteria for non-arteritic anterior ischemic optic neuropathy.
- Acute painless visual loss with normal optic discs and delayed visual evoked potentials should prompt consideration of retrobulbar optic neuropathy with a broad differential diagnosis.
- Markedly delayed visual evoked potential latency does not reliably distinguish between demyelinating and ischemic optic neuropathies in atypical cases.
- Patients with multiple vascular risk factors receiving GLP-1 receptor agonists should be counselled to promptly report new visual symptoms and undergo early ophthalmologic evaluation.
- Individual case reports contribute to pharmacovigilance but should be interpreted cautiously, as they do not establish causality.

## Contributors

All authors made substantial contributions to this work. SC was involved in patient management, conceptualization, supervision, and manuscript review. RDG and VC were involved in ophthalmologic evaluation, diagnostic interpretation, and critical manuscript revision. SP contributed to literature review, data collection, drafting of the manuscript, and formatting in accordance with journal requirements. All authors reviewed and approved the final version of the manuscript.

## Data Availability

Original data generated and analyzed during this study are included in this published article.
