# Correction to: “Atypical retrobulbar optic neuropathy after semaglutide escalation”

**DOI:** 10.1210/jcemcr/luag179

**Published:** 2026-07-06

In the above-named article by Channabasappa S, Das Gupta R, Chandran V, and Prasad S (JCEM Case Reports. 2026, 4(7); doi: 10.1210/jcemcr/luag108), there was an error in the Abstract and Case presentation sections.

In the originally published article, in the Abstract, in the first paragraph, the third sentence was originally published as, “We report a man in his early 30 seconds with class III obesity, obstructive sleep apnea, and prediabetes who developed acute, painless, asymmetric bilateral visual dysfunction four weeks after semaglutide escalation to 1 mg per week.” In the corrected article, “30 seconds” has been updated to “30s” and the sentence now reads, “We report a man in his early 30s with class III obesity, obstructive sleep apnea, and prediabetes who developed acute, painless, asymmetric bilateral visual dysfunction four weeks after semaglutide escalation to 1 mg per week.”

Similarly, in the originally published article, in the Case presentation, in the first paragraph, the first sentence was originally published as, “A man in his early 30 seconds with class III obesity (body mass index 47 kg/m2), severe obstructive sleep apnea managed with continuous positive airway pressure therapy, prediabetes, and dyslipidemia presented for medical management of obesity after unsuccessful lifestyle interventions.” In the corrected article, “30 seconds” has been updated to “30s” and the sentence now reads, “A man in his early 30s with class III obesity (body mass index 47 kg/m2), severe obstructive sleep apnea managed with continuous positive airway pressure therapy, prediabetes, and dyslipidemia presented for medical management of obesity after unsuccessful lifestyle interventions.”

The publisher regrets this error.

The article has been corrected online.

doi: https://doi.org/10.1210/jcemcr/luag108

